# Effects of phosphodiesterase type 5 inhibitors on choroid and ocular vasculature: a literature review

**DOI:** 10.1186/s40942-020-00241-0

**Published:** 2020-08-06

**Authors:** Natasha Ferreira Santos da Cruz, Murilo Ubukata Polizelli, Laís Maia Cezar, Emmerson B. Cardoso, Fernando Penha, Michel Eid Farah, Eduardo B. Rodrigues, Eduardo A. Novais

**Affiliations:** 1 Department of Ophthalmology, Federal University of São, Paulo Rua Botucatu 816, São Paulo, SP 04023-062 Brazil; 2 Department of Ophthalmology, HOlhos São Gonçalo, São Gonçalo, Brazil; 3 Department of Ophthalmology, Saint Louis University, St. Louis, MO USA

**Keywords:** Choroidal thickness, Enhanced depth imaging, Optical coherence tomography, PDE5 inhibitors

## Abstract

To provide information on the effects of phosphodiesterase type 5 (PDE5) inhibitors on choroidal vessels and central serous chorioretinopathy (CSC) and possible implications for development of exudative age-related macular degeneration (AMD). Two independent investigators conducted a qualitative review of PubMed to identify studies on the choroidal effect of PDE5 inhibitors in June 2019. The search used key words that included PDE5 inhibitors, sildenafil, tadalafil, vardenafil, choroid, choroidal flow, choroidal vessels, choroidal thickness, CSC, AMD or a combination. Only studies which assessed choroidal findings were included. Many ocular diseases are related to changes in choroidal thickness and perfusion. Patients with AMD, who have decreased choroidal perfusion, may manifest more severely diminished choroidal ability to deliver oxygen and other metabolites to the retina, leading to growth of neovascular tissue. As a result of this engorgement of the choroidal vasculature, some patients may have leakage across the retinal pigment epithelium (RPE) and accumulation of subretinal fluid, resulting in CSC. Transient visual symptoms, i.e., changes in color perception and increased light sensitivity, are well-known adverse effects, but there have been rare reports of vision-threatening ocular complications in users of PDE5 inhibitors, such as nonarteritic anterior ischemic optic neuropathy and cilioretinal artery occlusion. The choroid is a vascular tissue analogous in many respects to the corpus cavernosum, and PDE5 inhibitors may increase the choroidal thickness and perfusion. While it is intuitively obvious that thickness of the choroid alone does not guarantee better choriocapillaris oxygenation, it is a reasonable step towards ameliorating ischemia. These drugs have numerous physiologic effects on the choroid related to blood flow, such as clinical consequences in CSC and AMD.

## Introduction

Sildenafil (Viagra, Pfizer Pharmaceuticals, Secaucus, NJ, USA), tadalafil (Cialis, Eli Lilly Medical, Indianapolis, IN, USA), vardenafil (Levitra, Bayer Pharmaceuticals, Whippany, NJ, USA), and avanafil (Stendra, Vivus, Inc., Campbell, CA, USA) are used widely to treat pulmonary arterial hypertension and are currently the first-line pharmacologic treatments for erectile dysfunction (ED). These drugs are selective cyclic guanosine monophosphate (cGMP)-dependent PDE5 inhibitors, which induce vasodilation by enhancing the smooth muscle relaxant effects of nitric oxide (NO) [7, 64, 70, 78]. The enhanced dilation of blood vessels and lacunar spaces in the corpus cavernosum leads to improved penile erection in patients with ED [114]. The widening of blood vessels inside the lungs also decreases the pulmonary blood pressure (BP) to the heart and improves its function, helping to treat pulmonary arterial hypertension [76].

The ocular adverse reactions of this drug class are often mild and related to color and brightness perception in 3–11% of men taking sildenafil 25–100 mg [27], 0.3–2% of those taking vardenafil [54, 59], and 0.1% of tadalafil users [16]. Although rare, some severe reactions may occur, i.e., nonarteritic anterior ischemic optic neuropathy (NAION) with attendant visual loss, cilioretinal artery occlusion, central retinal vein occlusion, pupil-sparing third nerve palsy and glaucoma [2, 9, 14, 27, 39, 46, 58, 69, 70, 87, 92, 99]. Quiram et al. [89] also reported a case of Viagra-associated serous macular detachment. Even though these complications have been reported, men who use PDE5 inhibitors appear to have vision-threatening complications at the same frequency as the general population [6, 12].

PDE5 inhibitors decrease systemic BP, which can potentially lead to decreased choroidal circulation [75] that is deleterious in patients who may have compromised choroidal circulation or be at risk of ocular ischemic conditions. Indeed, several cases of NAION have been reported in patients taking sildenafil [15, 23, 29, 88]. In 2005, 43 cases of NAION in patients taking PDE5 inhibitors were reported to the Food and Drug Administration Adverse Event Reporting System [30]. Sildenafil was reported the most often, with 38 cases versus four cases for tadalafil and one case for vardenafil [6].

The possible effect of sildenafil in the choroid remains unknown. The current report reviews published studies that examined the effects of PDE5 inhibitors on the choroidal vessels, CSC, and their possible implications for exudative AMD, diseases that are associated with changes in choroidal thickness and perfusion.

## Materials and methods

### Literature search and selection

We conducted a qualitative review of the current literature pertaining to the effect of PDE5 inhibitors on the choroid. Two independent investigators searched and verified the PubMed findings in June 2019. A broad search strategy with appropriate descriptors and key words was used in PubMed that included PDE5 inhibitors, sildenafil, tadalafil, vardenafil, choroid, choroidal flow, choroidal vessels, choroidal thickness, CSC, AMD or a combination of them. All studies containing these descriptors were evaluated, but only those with choroidal findings were included. The articles were selected regardless of the moment of imaging or the time of previous drug use. No language or date restrictions were entered. Reference lists of identified primary reports and review articles were searched. Articles were reviewed and discussed among the authors, and relevant papers were then considered in detail.

## Results and discussion

### Pharmacology of PDE5 inhibitors

PDE5 inhibition increases the level of cGMP, an intracellular messenger affecting vasodilation by relaxation of smooth muscle in the arterioles. Production of cGMP from guanosine triphosphate is mediated through the NO signaling pathway. PDE5 inhibitors increase the cGMP levels, thereby potentiating the NO-elicited effect on sinusoidal vessels of the corpus cavernosum [43, 56].

PDE5 is largely expressed in the corpora cavernosa of the penis, but many other organs such as the pulmonary and coronary vasculature, sympathetic nervous system, and Purkinje neurons express the PDE5 enzyme, where its effect is less known [68, 91, 107]. PDE5 also directly affects the dilation of the retinal and choroidal vessels (smooth muscle and endothelial cells) and the ganglion (III neuron) and bipolar cell layers (II neuron). This is noteworthy because ganglion and bipolar cell layers act as filters in the visual signals and provide a first codification of the neural signal [24, 37, 81, 82, 102]. Other studies also have reported that PDE5 inhibitors significantly increase choroidal blood flow (CBF) [53, 61, 84, 96, 105].

**Fig. 1:**
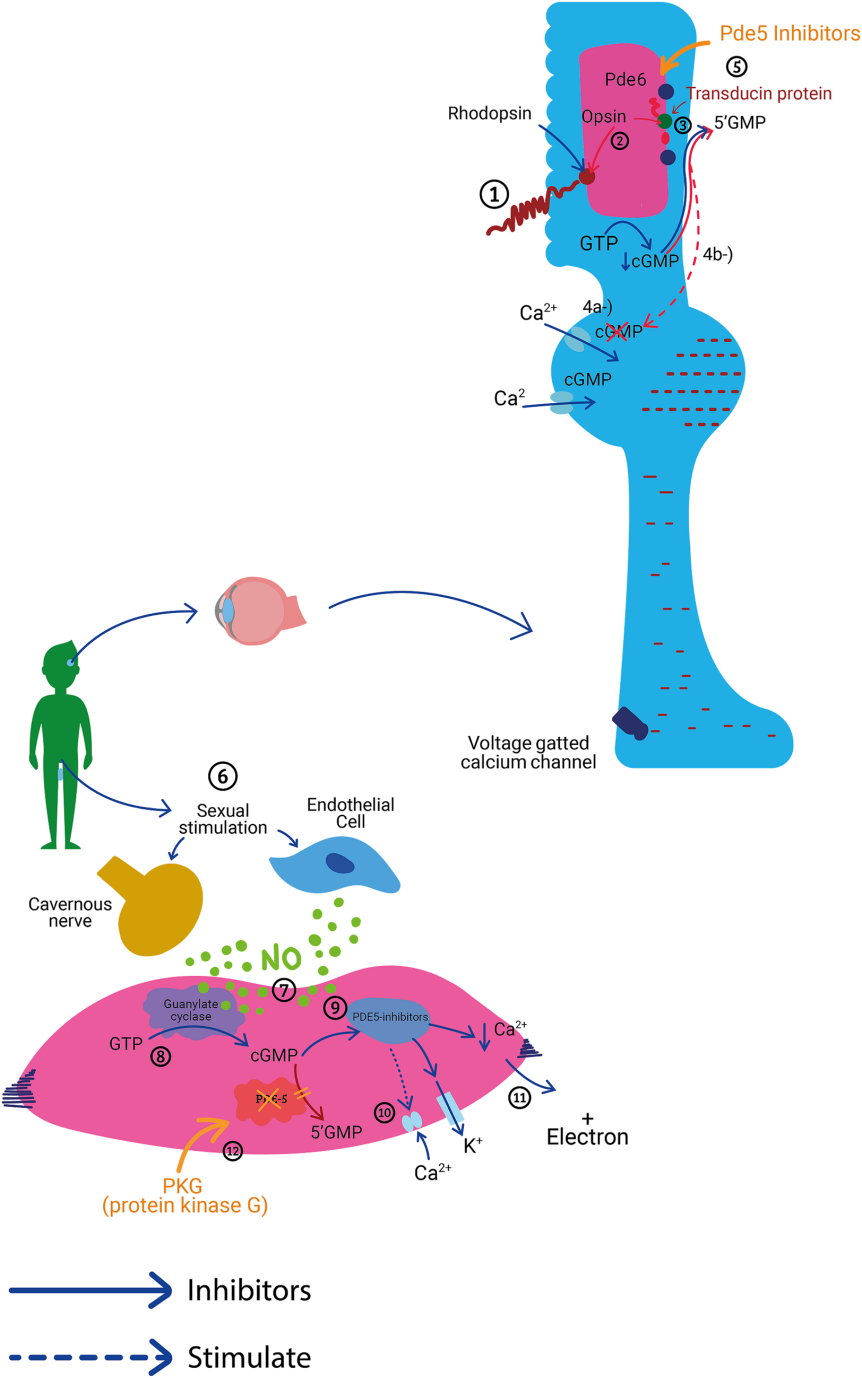
Mechanism of action of phosphodiesterase type 5 (PDE5) inhibitors: 1, light rays stimulate rhodopsin with 11-cis retinal; 2, opsin separates from all-trans-retinal and activates transducin protein; 3, transducin activates PDE6; 4, PDE6 hydrolyzes cyclic guanosine monophosphate (cGMP) into 5′GMP; a, cGMP binds to Ca^2+^ channels, stimulating Ca^2+^ uptake and b, when cGMP is hydrolyzed, these channels close and the cell is hyperpolarized, starting the neurotransmission; 5, PDE inhibitor stops cGMP hydrolysis, leaving Ca^2+^ channels open, and there is no hyperpolarization; 6, sexual stimulation causes nitric oxide (NO) production by nerve and endothelial cells; 7, NO mediates guanylate cyclase; 8, Guanylase cyclase (GC) transforms GTP (guanosine triphosphate) into cGMP. 9, protein kinase G (PKG) is activated by cGMP; 10, this cascade hyperpolarizes the cell by inhibiting Ca^2+^ uptake and stimulating K^+^ efflux; 11, the decrease in intracellular Ca^2+^ results in relaxation and penis erection; and 12, PDE5 hydrolyzes cGMP into 5′-GMP to stop the cycle. The PDE5 inhibitor has the same base of the cGMP entering the PDE5 receptor

These drugs also weakly inhibit PDE6 (Phosphodiesterase type 6), reflecting an affinity for PDE6 one-tenth that of PDE5 [7]. Because PDE6 is present in high concentrations in cone and rod cells and plays a key role in retinal light signal phototransduction [5, 17, 44, 110], its partial inhibition may account for the visual effects, such as impaired color discrimination and depressed scotopic responses [65, 106, 108] observed in flexible-dose controlled clinical trials of sildenafil in men with ED [22, 57, 70, 77, 97, 106] Fig. 1.

### Systemic and ocular effects

The standard recommended doses of Viagra range from 25 to 100 mg three times weekly. The peak plasma level after oral intake occurs at about 60 min with an elimination half-life of 3–5 h [34, 35]. The systemic side effects have been reported with an incidence of 2% or greater at doses of sildenafil up to 100 mg and include headache, flushing, dyspepsia, nasal congestion, urinary tract infection, diarrhea, dizziness, and rash [31–34]. Subjects also have been reported to have a 10-mmHg decrease in systemic BP [115]. Visual symptoms, including blue-tinted vision and/or increased light sensitivity, have been reported in 3% of men taking 25 mg of the drug, 11% taking 100 mg, and almost 50% taking 200 mg [36].

### Choroidal effect

The choroid, which supports the metabolic function of the outer retina, has been described as an erectile tissue, analogous to the corpus cavernosum. The fenestrated choroidal vasculature is highly responsive to local and neurogenic stimuli, and the uveal system may hold up to 97% of the intraocular blood volume [84].

Theoretically, because sildenafil has a strong systemic vasodilating effect that decreases systemic BP, this can result in decreased CBF [60]. However, since the choroid is similar to the corpus cavernosum, sildenafil could have a strong vasodilatory effect resulting in increased CBF as the result of a direct effect on the smooth muscle relaxation in the choroidal vessel walls [84]. The small variable effects we observed may be due to a different balance of these factors among individuals.

**Fig. 2:**
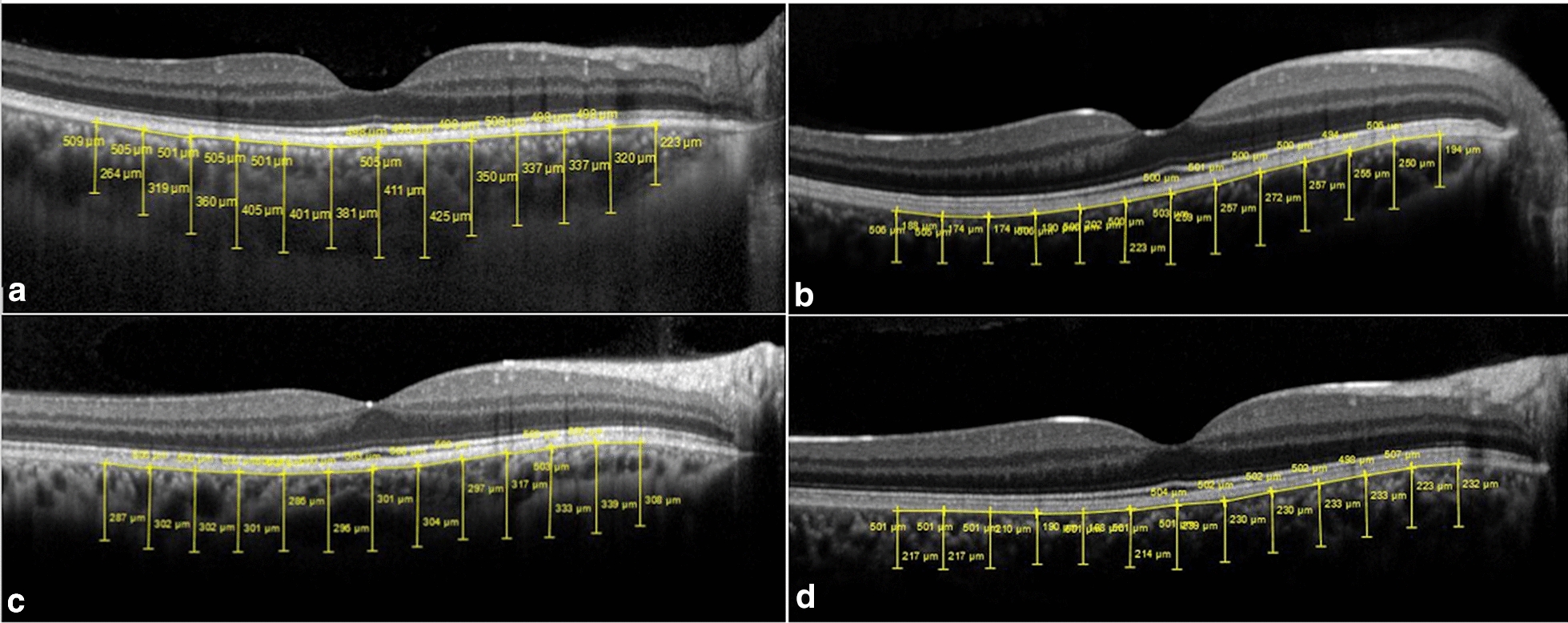
Comparison between enhanced depth imaging-optical coherence tomography scans in two patients of different ages matched for gender and age with controls. **A** A 42-year-old man used 100 mg sildenafil and had a subfoveal choroidal thickness of 505 mm. **B** A 34-year-old woman used 25 mg sildenafil and had a subfoveal choroidal thickness of 301 mm. **C** A 42-year-old man not taking any medication had a subfoveal choroidal thickness of 209 mm. **D** A 34-year-old woman not taking any medication had a subfoveal choroidal thickness of 239 mm

McCulley et al. [73], who assessed the choroidal thickness with ultrasonography with ultrasonography to correlate it with color vision and contrast sensitivity changes in a group of healthy subjects, did not report a consistent increase in the choroidal thickness after administration of 200 mg of sildenafil, a dose that is twice that of the highest recommended dose for treating ED. The study found only one subject who experienced a marked increase of 33% in the choroidal thickness, suggesting that some individuals could be especially responsive to sildenafil therapy. However, when the choroidal thickness was measured by enhanced-depth imaging-optical coherence tomography (EDI-OCT), the significant increases varied from 9.3 to 12.3% 1–3 h after ingesting 50–100 mg of sildenafil. This difference is likely due to low-resolution ultrasonography with small choroidal measurements compared to OCT, which has an approximate resolution of 5 microns. Other studies, however, showed no significant difference between the 1- and 3-h time points [60, 104] Fig. 2.

Interestingly, another study investigated the histopathologic effect of chronic sildenafil citrate use on the choroidal circulation in male rats and found a significant increase in the choroidal vascular dilation and congestion [105]. However, Vance et al. [104] noticed that choroidal vessels’ caliber changes after sildenafil assessed by EDI-OCT were not consistent for all study participants.

### Vascular effects

Most studies have reported increased CBF, with a lesser effect on the retinal vasculature. This differential effect may be due to differences in vascular innervation. Choroidal vessels are innervated by the autonomic nervous system, while innervation of the retinal vasculature is limited to the central retinal artery (CRA) posterior to the lamina cribrosa [10, 11, 66, 67]. In this respect, the innervation of the choriocapillaris resembles that of the corpus cavernosum. Nitric oxide triggers cGMP within smooth muscle cells as part of the signal transduction pathway in neural control of the choroidal vasculature, as it does in the vascular bed of the corpus cavernosum [84].

The retinal vessel endothelium continuously produces NO, which maintains the retinal vessels in a constantly dilated state [52]. Functionally, the retinal arteries and veins act as arterioles and venules. Because vascular resistance is correlated inversely with the fourth power of the radius of a blood vessel, small changes in diameter have a substantial effect on blood flow through the vessel by affecting the blood flow velocity in the ophthalmic artery (OA) and choroidal and retinal flow [26]. Therefore, the increase in retinal vessel diameter should lead to a considerable increase in the retinal blood flow (RBF) if the blood velocity is assumed to be constant [83].

### Techniques to assess vascularization and perfusion

Several studies have focused on the effects of sildenafil on the RBF and retinal vessel diameter in normal subjects. Despite these apparent discrepancies, this may result from different methods, techniques and/or sensitivities in measuring ocular blood velocity/flow, vascular diameter, or different medication times and doses.

Color Doppler image (CDI), one method to analyze the speed and direction of blood flow, showed significant increases in the OA peak systolic velocity (PSV) and the end diastolic velocity (EDV). Parallel increases in PSV and EDV can be interpreted as increased volumetric blood flow within a vessel [95].

CDI was used in some studies to elucidate the effect of sildenafil on blood flow. Dundar et al. [28] showed an increase in flow in the OA caused by 50 mg of sildenafil but failed to show increased flow in the CRA or temporal short posterior ciliary artery (SPCA), since it is technically difficult to locate and assess the SPCA using CDI.

Kurtulan et al. [63] noted that although sildenafil increased the mean cavernous artery PSV and also reduced systemic systolic and diastolic BP, it had no effect on CRA circulation even in subjects with ocular side effects. The alterations in choroidal perfusion and sildenafil-mediated inhibition of PDE6 were associated with ocular symptoms.

In a study similar to that of Kurtulan et al. [63], Koksal et al. [61] reinforced that sildenafil caused a significant increase in blood flow in the OA in a time-dependent manner, probably as a result of PDE5 inhibition on smooth muscle cells. Once again, vessels associated with the choroidal vasculature showed significantly greater volumetric blood flow when treated with sildenafil, while no effect was observed in the CRA.

In 2008, Foresta et al. reported that tadalafil and sildenafil modified the OA flow in a time-dependent manner. The administration of sildenafil increased PSV and EDV in the OA 60 min after administration. After 4 h, OA blood-flow velocity no longer differed from baseline. Tadalafil increased the PSV and EDV in the OA for at least 48 h. This result was consistent with the drug half-life (4 h for sildenafil and 48 h for tadalafil), so the modifications are not drug-specific but class-specific.

Taner et al [98], also using CDI, studied the effect of 50 mg of sildenafil on the retrobulbar and systemic hemodynamics during postural changes in healthy volunteers. However, no change was found in the OA, CRA, and SPCA blood-flow velocities between subjects taking 50 mg of sildenafil and controls.

Later in 2016, Matieli et al. [72] found no effects on blood flow in the OA, CRA, and ciliary arteries in patients who used sildenafil chronically for pulmonary arterial hypertension, when compared to controls. These results corroborated Taner et al.’s previous findings [98].

Sponsel et al. [96] and Paris et al. [84] used Heidelberg retina flowmetry (HRF) to assess the retina and pulsatile ocular blood flowmetry to evaluate the choroid flow.

HRF provides a two-dimensional map of blood flow to the optic nerve and surroundings of the retina. This technique, however, is most sensitive to blood flow changes in the superficial layers and therefore provides only limited information about deeper regions, which limits the ability to account for the RBF that is supplied by the choriocapillaris from the uveal system [8]. The authors found no changes in retinal capillary blood flow measured with the HRF and did not detect any change in the systemic BP.

Pulsatile ocular blood flow (POBF) determines the real‐time changes in ocular volume based on real‐time measurement of intraocular pressure. Most blood flow in the eye is in the choroidal circulation; therefore, it is presumed that POBF primarily measures the pulsatile component of the choroidal perfusion independent of the retinal or retrobulbar circulation [55]. Those authors found significant increases in the POBF in patients taking sildenafil that likely represents an increase in choroidal hemodynamics, though it is impossible to state with certainty the source of the changes in the ocular pulsatility. Since the CBF accounts for about 85% of the total ocular blood flow, significant changes in the POBF may be due to changes in the choroidal circulation. These data suggest that sildenafil produces an increased CBF [84, 96].

Laser Doppler Flowmetry (LDF) measures the blood flow in the capillary beds with the laser directed at areas between the larger vessels [8]. Grunwald et al. [51] examined the effects of sildenafil on blood flow in the optic nerve head and the foveolar region, where choroidal measurements were limited to the foveal avascular region of the retina. They investigators concluded that there were no changes in the CBF and optic nerve blood flow parameters after sildenafil, which is inconsistent with other studies. It is possible that limiting assessment of the choroidal vasculature to the macular avascular window may not be representative of the overall CBF, thus accounting for this inconsistency. The absence of changes in the optic nerve head blood flow may be interpreted as no change in the retinal hemodynamics, which agrees with Paris et al. [84].

Likewise, Metelitsina et al. [75] found no change in the CBF, despite a decrease in the systemic BP after administration of sildenafil. Those authors concluded that PDE5 inhibitors do not alter daytime ocular autoregulation. However, the same group later detected a significant increase in venous diameter up to 300 min after administering PDE5 inhibitors, using a more sensitive vessel measurement technique, the Vessel Map analysis program (IMEDOS GmbH, Weimar Germany) [74].

Polak et al. [86] also examined the effects of sildenafil on the retinal hemodynamics using laser Doppler flowmetry, the Retinal Vessel Analyzer (RVA) (Carl Zeiss Meditech, Dublin, CA) and response to flicker stimulation. The study showed a significant (15.7%) increase in RBF after 100 mg of sildenafil citrate and did not find flicker-induced retinal vasodilation.

Pache et al. [83] measured retinal arterial and venous diameters using the RVA after administration of 50 mg of sildenafil and reported increased vessel diameters, followed by a gradual decline toward baseline values during the following 90 min, suggesting an increase in RBF.

These data conflict with the findings of Paris et al. [84] and Grunwald et al. [51] and could have resulted because the latter studies were performed at the retinal capillary level, where the amount of increase in vessel diameter could have resulted in an undetectable increase in the blood flow level. Besides, the increase in vascular diameter could not be accompanied by a decrease in the retinal blood velocity, resulting in no change in the volumetric blood flow that could occur with increased CBF.

Polak et al. [86] also reported a 4.7% increase in retinal venous diameter using the RVA. As explained previously, this study used the results of both measures—diameter and velocity—to calculate the blood flow. These findings, combined with those of previous studies, suggest that sildenafil may have a slight effect on the retinal vasculature.

Using digitized fundus photographs, Grunwald et al. [49] examined the effect of sildenafil on retinal artery and vein diameters in a static way. Unlike the study of Pache et al. [83], no significant difference from baseline or placebo was detected in any vessel diameter, possibly because the RVA in the study of Pache et al. used a series of frames in a video that allowed for better resolution and details.

Diameter measurements, as with the RVA, achieves high resolution by performing numerous analyses at the frame rate of 25 Hz, meaning 25 images per second. As mentioned previously, Metelitsina et al. [74] improved their methods of analysis and increased the sensitivity of the technique to detect increased venous diameter compared to previous studies.

Swept-mode high-frequency digital ultrasound is a modification of the standard B-mode acquisition process that allows time-domain (as opposed to Doppler) assessment of flow by speckle tracking, as previously described [62, 93]. Speckle tracking is advantageous in maintaining high spatial resolution compared to color-flow Doppler. Kim et al. [60] used this technique and reported increased choroidal perfusion in 11 of 12 eyes, after the use of systemic sildenafil citrate.

Using a videomicroscopy technique, Yuan et al. [113] isolated, cannulated, and pressurized cadaveric porcine retinal arterioles without flow and reported that sildenafil caused modest arterial dilation in a dose-dependent manner. The authors emphasized that clinical doses of sildenafil did not cause substantial vasodilation and that the threshold concentration for sildenafil to dilate retinal arterioles was 10 ng/mL. The authors also found that the highest concentration (1 µg/mL) produced up to 30% maximal dilation, which has a notable impact on local retinal perfusion.

### Implications of PDE5 inhibitors on chorioretinal diseases

#### CSC

Serous detachment of the neurosensory retina can occur due to any process that disrupts the outer blood-ocular barrier controlled by the RPE [109]. The finding of focal leaks in the RPE together with impaired fluid reabsorption is characteristic of idiopathic CSC [112]. This subretinal leakage occurs due to altered choroidal vascular perfusion that leads to localized vascular congestion and impaired circulation, resulting in ischemia and facilitating choroidal exudation through a focally hyperpermeable choroid. Accordingly, the integrity of the choroidal vasculature has an important impact on subretinal fluid accumulation and plays a paramount role in normal retinal adhesion [19].

As demonstrated previously, PDE5 inhibitors increase the CBF and choroidal thickness [104], and as a result of engorgement of the choroidal vasculature, some patients may have leakage across the RPE and accumulated subretinal fluid [89]. The consequence is distorted or loss of central vision, decreased color perception, and relative scotoma [100].

Information about CSC has emerged during the use of sildenafil citrate for from 1 day to 2 years, so it is impossible to ascertain which dose of sildenafil citrate causes CSC at a specific time [25].

Murata et al. [79] was the first to report a unilateral case of CSC in a 33-year-old man taking sildenafil. The condition resolved within days when sildenafil was stopped (positive dechallenge) and recurred when he used sildenafil again a year later (positive rechallenge). After that case, many other unilateral and bilateral case have been described, in which some had a positive dechallenge with discontinuation of PDE5 inhibitors [1, 3, 4, 21, 38, 45, 80, 89, 90, 103], and some a positive rechallenge with PDE5 inhibitors [38, 89], proving that cessation of therapy was not associated with improved CSC in every case. Another important consideration is that CSC can resolve spontaneously, and the positive dechallenge may simply represent this possible outcome [38].

However, in a study of 43 prospective patients using sildenafil citrate, Damar et al. [25] did not identify one case of CSC. In 2010, French and Margo [40] reported a case-controlled postmarketing surveillance study that found that patients with CSC had no greater increase in prescription exposure to PDE5 inhibitors than did age-matched control subjects.

Although PDE5 inhibitors are classified as only a possible risk for CSC for suspected adverse drug reactions, it may be advisable to promote drug cessation in these cases, because choroidal congestion seems to play a role in the disorder and that any recurrence is possible if the therapy is resumed [38]. In addition, patients with serous macular detachments of unknown etiology should be questioned about their use of PDE5 inhibitors.

#### AMD

Many ocular diseases are related to changes in choroidal thickness and perfusion, including AMD [42, 71, 94, 101]. Grossniklaus and Green [47] postulated that for some patients decreases in the choroidal ability to deliver oxygen and other metabolites to the retina can lead to growth of neovascular tissue. In turn, this novel tissue “recapitulates the choriocapillaris and, theoretically, provides nutrients and oxygen to an ischemic RPE/outer retina that is expressing vascular endothelial grow factor” [47], and therefore, a thin choroid may not deliver the necessary nutrients and oxygen. Many authors have reported that patients with decreased choroidal perfusion showed increased severity of AMD [13, 18, 41, 48, 50, 85].

As mentioned previously, choroidal perfusion and thickness increase in response to systemic sildenafil [60]. While it is intuitively obvious that the choroidal thickness alone does not guarantee better choriocapillaris oxygenation, this could be a reasonable step toward ameliorating ischemia in dry AMD. Systemic treatment using a PDE5 and PDE6 inhibitor (sildenafil) is suggested as a means of increasing choroidal perfusion. Based on the hypothesis of increasing choroidal perfusion as a means of elevating NO (a messenger molecule) transfer across Bruch’s membrane by PDE5 as well as the increase in photoreceptor regeneration caused by a decrease in Warburg glycolysis by PDE6, some studies treated a series of patients with AMD with systemic sildenafil [20].

In a double-blinded phase II study, Birch et al. [12] examined the acute effect of sildenafil administration in patients with early AMD and showed no significant or clinically relevant changes in visual acuity, Humphrey perimetry, D15 color discrimination, photo-stress tests, and the Amsler grid. Furthermore, sildenafil did not cause significant changes in the foveolar choroidal circulation of patients with AMD [75] and showed similar vasodilatation of the major retinal veins as in normal subjects [74].

Coleman et al. [20] conducted a 2-year trial to evaluate the effect of sildenafil measured by spectral-domain OCT, color fundus photography, EDI, and best-corrected visual acuity. The group concluded that sildenafil is a safe treatment for AMD or vitelliform macular degeneration and suggested that a thickened Bruch’s membrane reduced the beneficial effect of the perfusion increase, but all eyes appeared to benefit from PDE6. The authors emphasized that the maintenance or improvement of the photoreceptor layer might have been the most significant result of sildenafil and that it was consistent with PDE6 inhibition. Thus, sildenafil treatment of macular degeneration offered significant potential for vision retention and recovery.

Nevertheless, given the lack of current evidence for the benefit and known cardiovascular and ocular risks of sildenafil, such as NAION, the use of PDE inhibitors as therapy for AMD should await further evaluation [111].

In conclusion, to date, PDE5 inhibitors have shown numerous effects on the choroid related to blood flow, such as clinical consequences in CSC and AMD. Based on their physiologic effects, PDE5 inhibitors could affect other diseases whose pathophysiology involves increased choroidal thickness. The treatment of other diseases with PDE5 inhibitors deserves further study. Thus, it is important to emphasize the importance of careful ophthalmologic follow-up in patients on this medication, so that possible consequences can be managed over time.

## Abbreviations

AMD: Age-related Macular Degeneration
BP: Blood Pressure
CBF: Choroidal Blood Flow
cGMP: Cyclic Guanosine Monophosphate
CRA: Central retinal artery
CSC: Central Serous Chorioretinopathy
DCI: Color Doppler image
ED: Erectile Dysfunction
EDI-OCT: Enhanced-Depth Imaging-Optical Coherence Tomography
EDV: End Diastolic Velocity
FDA: Food and Drug Administration
HRF: Heidelberg Retina Flowmetry
LDF: Laser Doppler Flowmetry
NAION: Nonarteritic Anterior Ischemic Optic Neuropathy
NO: Nitric Oxide
OA: Ophthalmic artery
PDE5: Phosphodiesterase type 5
PDE6: Phosphodiesterase type 6
POBF: Pulsatile Ocular Blood Flow
PSV: Peak Systolic Selocity
RBF: Retinal Blood Flow
RPE: Retinal Pigment Epithelium
RVA: Retinal Vessel Analyzer
SPCA: Short Posterior Ciliary Artery

## References

[1] Acar U, Kucuk B, Agin A, Koc M, Sobaci G (2014). Case report of tadalafil-induced central serous chorioretinopathy. J Clin Exp Ophthalmol.

[2] Akash R, Hrishikesh D, Amith P, Sabah S (2005). Case report: association of combined nonarteritic anterior ischemic optic neuropathy (NAION) and obstruction of cilioretinal artery with overdose of Viagra. J Ocular Pharmacol Ther.

[3] Aliferis K, Petropoulos IK, Farpour B, Matter MA, Safran AB (2012). Should central serous chorioretinopathy be added to the list of ocular side effects of phosphodiesterase 5 inhibitors?. Ophthalmologica.

[4] Allibhai ZA, Gale JS, Sheidow TS (2004). Central serous chorioretinopathy in a patient taking sildenafil citrate. Ophthalmic Surg Lasers Imaging.

[5] Arshavsky VY, Lamb TD, Pugh EN (2002). G proteins and phototransduction. Annu Rev Physiol.

[6] Azzouni F, Abu Samra K (2011). Are phosphodiesterase type 5 inhibitors associated with vision-threatening adverse events? A critical analysis and review of the literature. J Sex Med.

[7] Ballard SA, Gingell CJ, Tang K, Turner LA, Price ME, Naylor AM (1998). Effects of sildenafil on the relaxation of human corpus cavernosum tissue in vitro and on the activities of cyclic nucleotide phosphodiesterase isozymes. J Urol.

[8] Besharse J (2011). The Retina and its disorders.

[9] Bella AJ, Brant WO, Lue TF, Brock GB (2006). Non-arteritic anterior ischemic optic neuropathy (NAION) and phosphodiesterase type-5 inhibitors. Can J Urol.

[10] Bill A, Sperber GO (1990). Control of retinal and choroidal blood flow. Eye.

[11] Bill A (1985). Some aspects of the ocular circulation. Friedenwald lecture. Invest Ophthalmol Vis Sci.

[12] Birch DG, Toler SM, Swanson WH, Fish GE, Laties AM (2002). A double-blind placebo-controlled evaluation of the acute effects of sildenafil citrate (Viagra) on visual function in subjects with early-stage age-related macular degeneration. Am J Ophthalmol.

[13] Boker T, Fang T, Steinmetz R (1993). Refractive error and choroidal perfusion characteristics in patients with choroidal neovascularization and age-related macular degeneration. Ger J Ophthalmol.

[14] Bollinger K, Lee MS (2005). Recurrent visual field defect and ischemic optic neuropathy associated with tadalafil rechallenge. Arch Ophthalmol.

[15] Boshier A, Pambakian N, Shakir SA (2002). A case of nonarteritic ischemic optic neuropathy (NAION) in a male patient taking sildenafil. Int J Clin Pharmacol Ther.

[16] Brock GB, McMahon CG, Chen KK, Costigan T, Shen W, Watkins V (2002). Efficacy and safety of tadalafil for the treatment of erectile dysfunction: results of integrated analyses. J Urol.

[17] Chabre M, Deterre P (1989). Molecular mechanism of visual transduction. Eur J Biochem.

[18] Chen JC, Fitzke FW, Pauleikhoff D, Bird AC (1992). Functional loss in age-related Bruch’s membrane change with choroidal perfusion defect. Invest Ophthalmol Vis Sci.

[19] Chon CH, Yao XY, Dalal R (1996). An experimental model of retinal pigment epithelial and neurosensory serous detachment. Retina.

[20] Coleman DJ, Lee W, Chang S (2018). Treatment of macular degeneration with sildenafil: results of a two-year trial. Ophthalmologica.

[21] Coscas F, Coscas G, Zucchiatti I, Bandello F, Soubrane G, Souïed E (2012). Optical coherence tomography in tadalafil-associated retinal toxicity. Eur J Ophthalmol.

[22] Cote R (2004). Characteristics of photoreceptor PDE (PDE6): similarities and differences to PDE5. Int J Impot Res.

[23] Cunningham AV, Smith KH (2001). Anterior ischemic optic neuropathy associated with viagra. J Neuroophthalmol.

[24] Dacey DM, Liao HW, Peterson BB, Robinson FR, Smith VC, Pokorny J (2005). Melanopsin-expressing ganglion cells in primate retina signal colour and irradiance and project to the LGN. Nature.

[25] Damar E, Toklu Y, Tuncel A, Balci M, Aslan Y, Simsek S, Atan A (2013). Does therapeutic dose of sildenafil citrate treatment lead to central serous chorioretinopathy in patients with erectile dysfunction?. Am J Mens Health.

[26] Delaey C, Van De Voorde J (2000). Regulatory mechanisms in the retinal and choroidal circulation. Ophthalmic Res.

[27] Donahue SP, Taylor RJ (1998). Pupil-sparing third nerve palsy associated with sildenafil citrate (Viagra). Am J Ophthalmol.

[28] Dundar SO, Dundar M, Kocak I, Dayanir Y, Ozkan SB (2001). Effect of sildenafil on ocular haemodynamics. Eye.

[29] Egan R, Pomeranz H (2000). Sildenafil (Viagra) associated anterior ischemic optic neuropathy. Arch Ophthalmol.

[30] Food and Drug Administration (FDA). FDA announces revisions to labels for Cialis, Levitra and Viagra: potential risk of sudden hearing loss with ED drugs to be displayed more prominently. US Food and Drug Administration Web site. 2005. http://www.fda.gov/NewsEvents/Newsroom/PressAnnouncements/2007/ucm109012.htm. Accessed 21 June 2009.

[31] Food and Drug Administration (FDA a) Joint Clinical Review. Study 101/101B. A randomized, double-blind, placebo-controlled, parallel-group, fixed-dose, multicenter, long-term dose-response study to assess the efficacy and safety of sildenafil (UK-92, 480) administered prior to sexual activity to male patients with erectile dysfunction. Silver Spring, MD: Food and Drug Administration; 1998. p. 99–102.

[32] Food and Drug Administration (FDA b) Joint Clinical Review. Study 148–102 (1998). A doubleblind, randomized, placebo controlled, parallel- group, fixed-dose, multicenter, study to assess the efficacy and safety of UK-92, 480 administered over six months to male patients with erectile dysfunction.

[33] Food and Drug Administration (FDA c) Joint Clinical Review. Study 148–103 (1998). A doubleblind, randomized, placebo-controlled, parallel-group, multicenter, flexible dose escalation study to assess the efficacy and safety of sildenafil administered as required to male patients with erectile dysfunction.

[34] Food and Drug Administration (FDA d) Joint Clinical Review. Study 148–004 (1998). Phase 1 investigator- blind, placebo-controlled evaluation of safety, toleration, and pharmacokinetics of sildenafil following escalating single oral doses in healthy male volunteers.

[35] Food and Drug Administration (FDA e) Joint Clinical Review. Study 148–228 (1998). An open, randomized, single oral dose, four way crossover study to determine the dose proportionality of the pharmacokinetics of sildenafil in healthy male volunteers over the dose range 15 mg to 200 mg.

[36] Food and Drug Administration (FDA f) Joint Clinical Review. Study 148–223 (1998). An open, randomized, placebo-controlled, four period crossover study to assess the effect of orally administered sildenafil (50, 100 and 200 mg) on visual function in healthy male volunteers.

[37] Foresta C, Caretta N, Zuccarello D, Poletti A, Biagioli A, Caretti L (2008). Expression of the PDE5 enzyme on human retinal tissue: new aspects of PDE5 inhibitors ocular side effects. Eye.

[38] Fraunfelder FW, Fraunfelder FT (2008). Central serous chorioretinopathy associated with sildenafil. Retina.

[39] Fraunfelder FW, Shults T (2006). Non-arteritic anterior ischemic optic neuropathy, erectile dysfunction drugs, and amiodarone: is there a relationship?. J Neuroophthalmol.

[40] French DD, Margo CE (2010). Central serous chorioretinopathy and phosphodiesterase-5 inhibitors: a case-control postmarketing surveillance study. Retina.

[41] Friedman E, Krupsky S, Lane AM, Oak SS, Friedman ES, Egan K (1995). Ocular blood flow velocity in age-related macular degeneration. Ophthalmology.

[42] Gemenetzi M, De Salvo G, Lotery AJ (2010). Central serous chorioretinopathy: an update on pathogenesis and treatment. Eye.

[43] Goldstein I, Lue TF, Padma-Nathan H, Rosen RC, Steers WD, Wicker PA (1998). Oral Sildenafil in the treatment of erectile dysfunction. N Engl J Med.

[44] Gonzalez CM, Bervig T, Podlasek C, Huang CF, McKenna KE, McVary KT (1999). Sildenafil causes a dose-and time-dependent downregulation of phosphodiesterase type 6 expression in the rat retina. Int J Impot Res.

[45] Gordon-Bennett P, Rimmer T (2012). Central serous chorioretinopathy following oral tadalafil. Eye.

[46] Gorkin L, Hvidsten K, Sobel RE, Siegel R (2006). Sildenafil citrate use and the incidence of nonarteritic anterior ischemic optic neuropathy. Int J Clin Pract.

[47] Grossniklaus HE, Green WR (2004). Choroidal neovascularization. Am J Ophthalmol.

[48] Grunwald JE, Hariprasad SM, DuPont J, Maguire MG, Fine SL, Brucker AJ (1998). Foveolar choroidal blood flow in age-related macular degeneration. Invest Ophthalmol Vis Sci.

[49] Grunwald JE, Metelitsina T, Grunwald L (2002). Effect of sildenafil citrate (Viagra) on retinal blood vessel diameter. Am J Ophthalmol.

[50] Grunwald JE, Metelitsina TI, Dupont JC, Ying GS, Maguire MG (2005). Reduced foveolar choroidal blood flow in eyes with increasing AMD severity. Invest Ophthalmol Vis Sci.

[51] Grunwald JE, Siu KK, Jacob SS (2001). Effect of sildenafil citrate (Viagra) on the ocular circulation. Am J Ophthalmol.

[52] Haefliger IO, Flammer J, Luscher TF (1992). Nitric oxide and endothelin-1 are important regulators of human ophthalmic artery. Invest Ophthalmol Vis Sci.

[53] Harris A, Kagemann L, Ehrlich R, Ehrlich Y, Lopez CR, Purvin VA (2008). The effect of sildenafil on ocular blood flow. Br J Ophthalmol.

[54] Hellstrom WJ, Gittelman M, Karlin G, Segerson T, Thibonnier M, Taylor T (2002). Vardenafil for treatment of men with erectile dysfunction: efficacy and safety in a randomized, double-blind, placebo-controlled trial. J Androl.

[55] Hitchings R (1991). The ocular pulse. Br J Ophthalmol.

[56] Ignarro LJ, Cirino G, Casini A, Napoli CJ (1999). Nitric oxide as a signaling molecule in the vascular system: an overview. Cardiovasc Pharmacol.

[57] Jagle H, Jagle C, Serey L, Yu A, Rilk A, Sadowski B (2004). Visual short-term effects of Viagra: double-blind study in healthy young subjects. Am J Ophthalmol.

[58] Johnson LN, Arnold AC (1994). Incidence of nonarteritic and arteritic anterior ischemic optic neuropathy. Population-based study in the state of Missouri and Los Angeles County California. J Neuroophthalmol.

[59] Kim CM, Kim YS, Sunwoo S, Cho B, Rho M, Yang YJ (2007). Post-marketing surveillance study of the efficacy and safety of vardenafil among patients with erectile dysfunction in primary care. Int J Impot Res.

[60] Kim DY, Silverman RH, Chan RV, Khanifar AA, Rondeau M, Lloyd H, Schlegel P, Coleman DJ (2013). Measurement of choroidal perfusion and thickness following systemic sildenafil (Viagra^®^). Acta Ophthalmol.

[61] Koksal M, Ozdemir H, Kargi S, Yesilli C, Tomac S, Mahmutyazicioglu K (2005). The effects of sildenafil on ocular blood flow. Acta Ophthalmol Scand.

[62] Kruse DE, Silverman RH, Fornaris RJ, Coleman DJ, Ferrara KW (1998). A swept-scanning mode for estimation of blood velocity in the microvasculature. IEEE Trans Ultrason Ferroelectr Freq Control.

[63] Kurtulan E, Gulcu A, Secil M (2004). Effects of sildenafil on ocular perfusiondemonstrated by color Doppler ultrasonography. Int J Impot Res.

[64] Laties A, Ellis P, Koppiker N, Patat A, Stuckey B (1998). Visual function testing in patients and healthy volunteers receiving VIAGRA. Ophthalmic Res.

[65] Laties A, Zrenner E (2002). Viagra (sildenafil citrate) and ophthalmology. Prog Retin Eye Res.

[66] Laties AM (1967). Central retinal artery innervation. Absence of adrenergic innervation to the intraocular branches. Arch Ophthalmol.

[67] Lieb WA, Bielfeld G (1967). Anatomy histology and physiology of capillary vessels of the eye fundus. Med Welt.

[68] Lin CS (2004). Tissue expression, distribution, and regulation of PDE5. Int J Impot Res.

[69] Lincoln TM (2004). Cyclic GMP and phosphodiesterase 5 inhibitor therapies: what's on the horizon?. Mol Pharmacol.

[70] Marmor MF, Kessler R (1999). Sildenafil (Viagra) and ophthalmology. Surv Ophthalmol.

[71] Maruko I, Iida T, Sugano Y, Oyamada H, Sekiryu T, Fujiwara T (2011). Subfoveal choroidal thickness after treatment of Vogt-Koyanagi-Harada disease. Retina.

[72] Matieli L, Berezovsky A, Salomão SR (2016). Ocular toxicity assessment of chronic sildenafil therapy for pulmonary arterial hypertension. Graefes Arch Clin Exp Ophthalmol.

[73] McCulley TJ, Luu JK, Marmor MF, Feuer WJ (2002). Effects of sildenafil citrate (Viagra) on choroidal congestion. Ophthalmologica.

[74] Metelitsina TI, Grunwald JE, DuPont JC, Ying GS, Liu C (2006). Effect of Viagra on retinal vein diameter in AMD patients. Exp Eye Res.

[75] Metelitsina TI, Grunwald JE, DuPont JC, Ying GS (2005). Effect of Viagra on the foveolar choroidal circulation of AMD patients. Exp Eye Res.

[76] Michelakis ED, Tymchak W, Noga M, Webster L, Wu XC, Lien D (2003). Long-term treatment with oral sildenafil in safe and improves functional capacity and hemodynamics in patients with pulmonary arterial hypertension. Circulation.

[77] Morales A, Gingell C, Collins M, Wicker PA, Osterloh IH (1998). Clinical safety of oral sildenafil citrate (VIAGRA) in the treatment of erectile dysfunction. Int J Impot Res.

[78] Moreland RB, Goldstein I, Traish A (1998). Sildenafil, a novel inhibitor of phosphodiesterase type 5 in human corpus cavernosum smooth muscle cells. Life Sci.

[79] Murata M, Ideta H, Kawasaki T, Noda Y (2000). A case of central serous chorioretinopathy after sildenafil (Viagra). Kyushu Ganka Gakkai.

[80] Nadeau S, Nguyen F, Guigou S (2012). Serous central chorioretinopathy and tadalafil: a case report. J Fr Ophtalmol.

[81] Neves G, Lagnado L (1999). The retina. Curr Biol.

[82] Nirenberg S, Carcieri SM, Jacobs AL, Lathman PE (2001). Retinal ganglion cells act largely as independent encoders. Nature.

[83] Pache M, Meyer P, Prunte C (2002). Sildenafil induces retinal vasodilatation in healthy subjects. Br J Ophthalmol.

[84] Paris G, Sponsel WE, Sandoval SS, Elliott WR, Trigo Y, Sanford DK (2001). Sildenafil increases ocular perfusion. Int Ophthalmol.

[85] Pauleikhoff D, Chen JC, Chisholm IH, Bird AC (1990). Choroidal perfusion abnormality with age-related Bruch’s membrane change. Am J Ophthalmol.

[86] Polak K, Wimpissinger B, Berisha F (2003). Effects of sildenafil on retinal blood flow and flicker-induced retinal vasodilatation in healthy subjects. Invest Ophthalmol Vis Sci.

[87] Pomeranz HD, Bhavsar AR (2005). Nonarteritic ischemic optic neuropathy developing soon after use of sildenafil (Viagra): a report of seven new cases. J Neuroophthalmol.

[88] Pomeranz HD, Smith KH, Hart WM, Egan RA (2002). Sildenafil-associated nonarteritic anterior ischemic optic neuropathy. Ophthalmology.

[89] Quiram P, Dumars S, Parwar B, Sarraf D (2005). Viagra-associated serous macular detachment. Graefe Arch Clin Exp Ophthalmol.

[90] Roy RI, Panigrahi P, Saurabh K, Das D, Lobo A (2014). Central serous chorioretinopathy following oral tadalafil intake. Clin Exp Optom.

[91] Schwarz ER, Kapur V, Rodriguez J, Rastogi S, Rosanio S (2007). The effects of chronic phosphodiesterase-5 inhibitor use on different organ systems. Int J Impot Res.

[92] Shindel AW (2009). 2009 update on phosphodiesterase type 5 inhibitor therapy part 2: updates on optimal utilization for sexual concerns and rare toxicities in this class. J Sex Med.

[93] Silverman RH, Kruse DE, Coleman DJ, Ferrara KW (1999). High-resolution ultrasonic imaging of blood flow in the anterior segment of the eye. Invest Ophthalmol Vis Sci.

[94] Spaide RF (2009). Age-related choroidal atrophy. Am J Ophthalmol.

[95] Spencer JA, Giussani DA, Moore PJ (1991). In vitro validation of Doppler indices using blood and water. J Ultrasound Med.

[96] Sponsel WE, Paris G, Sandoval SS, Sanford DK, Harrison JM, Elliott WR (2000). Sildenafil and ocular perfusion. N Engl J Med.

[97] Stockman A, Sharpe LT, Tufail A, Kell PD, Jeffery G (2006). Viagra slows the visual response to flicker. Curr Biol.

[98] Taner P, Ergin A, Basar MM (2005). Sildenafil does not alter retrobulbar hemodynamics in postural variations. Neuroophthalmology.

[99] Thurtell MJ, Tomsak RL (2008). Nonarteritic anterior ischemic optic neuropathy with PDE-5 inhibitors for erectile dysfunction. Int J Impot Res.

[100] Tittl MK, Spaide RF, Wong D (1999). Systemic findings associated with central serous chorioretinopathy. Am J Ophthalmol.

[101] Torres VL, Brugnoni N, Kaiser PK, Singh AD (2011). Optical coherence tomography enhanced depth imaging of choroidal tumors. Am J Ophthalmol.

[102] Trexler EB, Li W, Massey SC (2005). Simultaneous contribution of two rod pathways to All amacrine and cone bipolar cell light responses. J Neurophysiol.

[103] Turkcu FM, Yuksel H, Sahin A, Murat M, Bozkurt Y, Caca I (2013). Central serous chorioretinopathy due to tadalafil use. Int Ophthalmol.

[104] Vance SK, Imamura Y, Freund KB (2011). The effects of sildenafil citrate on choroidal thickness as determined by enhanced depth imaging optical coherence tomography. Retina.

[105] Vatansever HS, Kayikcioglu O, Gumus B (2003). Histopathologic effect of chronic use of sildenafil citrate on the choroid & retina in male rats. Indian J Med Res.

[106] Vobig MA, Klotz T, Staak M, Bartz-Schmidt KU, Engelmann U, Walter P (1999). Retinal side effects of sildenafil. Lancet.

[107] Waldkirch E, Uckert S, Yildirim H, Sohn M, Jonas U, Stief CG (2005). Cyclic AMP-specific and cyclic GMP-specific phosphodiesterase isoenzymes in human cavernous arteries—immunohistochemical distribution and functional significance. World J Urol.

[108] Wallis RM (1999). The pharmacology of sildenafil, a novel and selective inhibitor of phosphodiesterase (PDE) type 5. Nippon Yakurigaku Zasshi.

[109] Wolfensberger TJ, Tufail A (2000). Systemic disorders associated with detachment of the neurosensory retina and retinal pigment epithelium. Curr Opin Ophthalmol.

[110] Yamazaki A, Moskvin O, Yamazaki RK (2002). Phosphorylation by cyclin-dependent protein kinase 5 of the regulatory subunit (Pgamma) of retinal cgmp phosphodiesterase (PDE6): its implications in phototransduction. Adv Exp Med Biol.

[111] Yiu G, Vuong VS, Tran S, Migacz J, Cunefare D, Farsiu S (2019). Vascular response to sildenafil citrate in aging and age-related macular degeneration. Sci Rep.

[112] Yoon YH, Marmor MF (1993). Retinal pigment epithelium adhesion to Bruch’s membrane is weakened by hemicholinium- 3 and sodium iodate. Ophthalmic Res.

[113] Yuan Z, Hein TW, Rosa RH, Kuo L (2008). Sildenafil (Viagra) evokes retinal arteriolar dilation: dual pathways via NOS activation and phosphodiesterase inhibition. Invest Ophthalmol Vis Sci.

[114] Zrenner E, Koppiker NP, Smith MD, Constable I, Littlewood R, Stuckey B (2000). The effects of long-term sildenafil treatment on ocular safety in patients with erectile dysfunction. Invest Ophthalmol Vis Sci.

[115] Zusman RM, Morales A, Glasser DB, Osteroh IH (1999). Overall cardiovascular profile of sildenafil citrate. Am J Cardiol.

